# The effect of seed priming on yield and germination properties of quinoa (*Chenopodium quinoa* Willd)

**DOI:** 10.1038/s41598-026-41546-8

**Published:** 2026-02-27

**Authors:** Parisa Ahmadi, Farzad Hosseinpanahi, Adel Siosemardeh, Bahman Fazeli-Nasab

**Affiliations:** 1 Department of Plant Production and Genetics, Faculty of Agriculture, University of Kurdistan, Sanandaj, 66177-15175 Iran; 2 Department of Agronomy and Plant Breeding, Agriculture Institute, Research Institute of Zabol, Zabol, Iran

**Keywords:** Germination, Hormonal Priming, Osmopriming, Quinoa yield, Seed priming, Physiology, Plant sciences

## Abstract

Seed priming is a widely used pre-germination technique that enhances sprouting efficiency, synchrony, and the initial development of plantlets, particularly under suboptimal environmental conditions. This study aimed to evaluate the impact of different seed priming treatments on the germination, growth, and yield performance of quinoa (Chenopodium quinoa Willd.) through a series of laboratory, greenhouse, and field experiments conducted during the 2024 cropping season. The laboratory experiment was arranged in a completely randomized design (CRD) with four replications. Treatments included potassium nitrate (5% w/w), potassium chloride (1% w/w), zinc sulfate (6% w/w), gibberellic acid (30 mg.kg⁻¹), polyethylene glycol 6000 (10% v/v), salicylic acid (200 mg. L⁻¹), humic acid (54 mg. L⁻¹), hydropriming (distilled water), and a non-primed control. The same treatments were evaluated in a greenhouse trial (CRD with four replications) and a field trial (randomized complete block design with three replications). The overall results of the first experiment showed that treatments of potassium chloride, hydropriming, potassium nitrate, and gibberellic acid had positive effects on the germination characteristics of quinoa seeds. The greenhouse experiment also clearly demonstrated the superiority of potassium chloride and gibberellic acid treatments. In the field experiment, salicylic acid and potassium chloride treatments led to the highest economic yield. Based on the combined results of the experiments in this study, priming quinoa seeds with potassium chloride and salicylic acid is recommended.

## Introduction

The global population is projected to reach about 9.8 billion by 2050 and 11.2 billion by 2100. This rapid growth necessitates a significant increase in food production to ensure global food security^1^. However, climate change—alongside population expansion—is increasingly compromising crop productivity by exerting stress on agricultural systems^2^. Rising temperatures negatively impact crop development by reducing the soil’s ability to retain water and maintain fertility. Unchecked population growth and ongoing climate change have worsened drought stress and accelerated the decline of soil fertility. To maintain agricultural productivity under these pressures, it is essential to prioritize climate-resilient crops. *C. quinoa* Willd., commonly known as quinoa, is a stress-tolerant pseudo-cereal with significant potential to meet global food demand and alleviate poverty in a warming world^3^. Its inherent tolerance to salinity and drought enables quinoa to substitute for more sensitive crops under future climate scenarios. Nonetheless, successful cultivation is constrained by small seed size, which complicates germination and seedling establishment^4^.

The global production rate of quinoa (Chenopodium quinoa Willd.) varies widely by country and environmental conditions^5^. In the Andean region, quinoa yields have been reported according to the region’s specific conditions^6^. Other quinoa-growing countries also show varied yields^5^. The global production trend of this crop over different years has been studied^7^. The share of different countries in global quinoa production and the significance of the Andean region as the primary cultivation area have also been confirmed^8^.

The production rate of quinoa (C. quinoa Willd.) in Iran varies depending on genotype, location, and cultivation conditions. Several studies report quinoa seed yield under diverse and often stressful Iranian climatic conditions, indicating a range of performance across different environments^9–11^. Research in various locations, including Karaj, Shahrekord, Ahvaz, and Iranshahr, has evaluated the performance of different genotypes, with some showing notably higher yields in trials^11^. Studies have also investigated the effects of sowing date and nitrogen fertilization on seed yield in Ahvaz^9^. Sowing date experiments in Yazd have contributed to understanding production potential in that region^10^.

Seed germination plays a fundamental role in seedling establishment, vigor, and overall plant health^12^. Traits such as uniform emergence and a high seedling emergence rate are key indicators of seed efficiency, directly influencing early plant development and stand establishment^13^. Seed priming improves quinoa germination by promoting faster and more synchronized sprouting. Seeds that germinate quickly move into the seedling phase sooner, allowing them to utilize environmental resources more effectively. This leads to better establishment and reduced susceptibility to abiotic stresses. As a result, seed priming is widely recommended to enhance germination performance and seedling vigor, ultimately improving early plant establishment^2^. By accelerating germination and promoting uniform emergence, priming contributes to enhanced seedling development, higher yields, and improved crop quality. Moreover, it represents a cost-effective strategy for increasing agricultural productivity by stimulating the accumulation of bioactive compounds during early growth stages^14^. Numerous studies have highlighted the benefits of seed priming across various crops. For instance, it has been demonstrated^15^ that priming significantly improved the leaf area index, 1000-seed weight, and overall yield of the *Titicaca* quinoa variety.

Research indicates that salinity stress caused by sodium chloride decreases quinoa seed germination. However, priming seeds with gibberellic acid can counteract this negative effect and improve germination rates^16^. Osmotic priming has also been reported to alleviate the adverse impacts of salt stress on germination traits, shoot length, and root length^17^. In addition, priming with chitosan and potassium nitrate has been shown to mitigate the metabolic costs associated with the synthesis of soluble sugars, proline, and antioxidant enzymes, thereby preserving dry matter accumulation in quinoa plants^18^. It has been studied^19^ the effects of salicylic acid priming on two quinoa hybrids (*Giza1* and *Titicaca*) under salt stress and found that elevated salinity levels reduced photosynthetic pigment content and germination indices. However, salicylic acid treatment improved germination percentage, seedling vigor, and longitudinal growth, ultimately supporting better plant establishment under saline conditions.

In a separate study, priming quinoa seeds with potassium nitrate was shown to increase germination percentage and rate, shoot length, chlorophyll a and b contents, carotenoid content, protein levels, and catalase enzyme activity, while reducing electrolyte leakage from the plasma membrane^20^. Similarly, it has been investigated^21^ the effects of hydropriming and biopriming with *Bacillus subtilis* on quinoa plants under drought stress. Their results showed that both treatments mitigated the negative effects of drought stress and improved traits like root dry weight, proline content, superoxide dismutase activity, and anthocyanin content. Furthermore, osmopriming and hormonal seed priming of rice using potassium nitrate and salicylic acid enhanced seed germination, plant growth, and antioxidant enzyme activities in drought-stress conditions^22^.

It has been reported^23^ that hormonal priming with salicylic acid helps maintain cytokinin and auxin levels, enhances germination, reduces average germination time, promotes plant cell division, and improves corn plant growth and yield. In a separate study, it has been demonstrated^24^ that hormonal priming with gibberellin, auxin, and cytokinin increased germination traits and seedling growth in *Bromus*, and additionally noted that gibberellin elevated chlorophyll content in these plants. Similarly, it has been found^25^ that hormonal priming with gibberellin and kinetin decreased the mean germination time and improved greening in wheat seeds. Furthermore, it has been reported^26^ that hydropriming of chickpea seeds significantly increased seedling length, seed vigor, germination percentage, and germination rate. Another study reported that priming treatments increased grain yield in sesame, with hydropriming exhibiting the most pronounced effects^27^.

Rapid and uniform germination and early seedling establishment are key factors in realizing a crop’s yield potential. In this regard, seed priming is recognized as a practical and effective strategy for improving germination indices and initial seedling vigor. While the positive effects of this method on quinoa seedling characteristics under controlled laboratory conditions have been well-documented, there is limited empirical evidence regarding the efficacy and sustainability of these effects under real field conditions, particularly in specific ecosystems such as the semi-arid climate of Iran. This knowledge gap highlights the need for comparative studies in both controlled and field environments. Therefore, the main objective of this research is to comparatively evaluate the effects of different seed priming treatments on germination indices in the laboratory, as well as on seedling establishment, growth, and final yield under field conditions in the climate of Kurdistan Province, Iran. The results of this study can help identify the most effective priming method for improving establishment and increasing the sustainable yield of field-grown quinoa under similar conditions.

## Materials and methods

The quinoa seeds (*Chenopodium quinoa* Willd.) used in this study are of cultivated origin and were provided by the Kurdistan Agricultural and Natural Resources Research and Education Center. The center has granted permission for the research use of these seeds in accordance with the guidelines on the collection of plant material, ensuring full compliance with all relevant institutional, national, and international guidelines and legislation. The experimental treatments included a control (without priming), hormonal priming with gibberellic acid (30 mg. L⁻¹), and salicylic acid (200 mg. L⁻¹), and various osmopriming treatments such as potassium chloride (1% w/v), potassium nitrate (5% w/v), polyethylene glycol 6000 (10% w/v), humic acid (54 mg.L^− 1^), zinc sulfate (6% w/v), and hydropriming with distilled water. The experiments were conducted on seeds of the quinoa variety “Titicaca.”

This study was conducted through three experiments:

- Experiment 1 (laboratory): seed priming test in Petri dishes.
- Experiment 2 (greenhouse): seed priming test in pots.
- Experiment 3 (field): seed priming test in field condition.

### Experiment 1 (Laboratory): Seed priming test in petri dishes

First To prepare for the priming treatments, all equipment and germination papers were sterilized in an autoclave at 121 °C for 20 min. Before priming, the seeds were surface-disinfected with 2% sodium hypochlorite for 5 min and rinsed several times with distilled water. Priming solutions were prepared by dissolving specific amounts of each priming agent in distilled water to the desired concentrations. Seeds were then immersed in the prepared solutions and incubated for 6 h at 21–22 °C, with continuous aeration provided by an air pump. After priming, seeds were dried at room temperature for 24 h before being transferred to the germination and cultivation medium.

The selected treatments were initially evaluated under controlled conditions using a completely randomized design for preliminary assessment of the priming effects. Four Petri dishes were used as replicates for each treatment, with 100 seeds placed on Whatman No. 1 filter paper^28^ in each dish and moistened with 5 ml of distilled water. The Petri dishes were then transferred to a germinator set at 20 ± 1 °C, with 70% relative humidity, and a photoperiod of 16 h light and 8 h darkness^28^.

The seeds were incubated in the germinator for ten days. During the experiment, the number of germinated seeds was recorded daily over one hour. Seed germination was defined by the emergence of a 2 mm root. Germination percentage, germination rate, and mean germination time were calculated on the seventh day, while shoot and root lengths were measured on the tenth day.

The following equations were used to calculate germination percentage, germination rate, and mean germination time based on the number of germinated seeds recorded at the end of the counting period^29^:

(1) FGP=(∑n)/*N*×100.

In this equation, ∑n is the total number of germinated seeds, and N is the number of cultivated seeds.

(2) R=∑ni/di.

(2) MGT=(∑ (nd))/(∑n).

In this equation, D is the time in days, n is the number of germinated seeds per day, and ∑n is the total number of germinated seeds. After the experiment, shoot and root lengths for each treatment were measured using a ruler.

### Experiment 2 (greenhouse): seed priming test in pots

The greenhouse experiment was conducted using a completely randomized design with four replications in the research greenhouse of Kurdistan University (latitude 35.19°N, longitude 46.59°E, altitude 1480 m above sea level). The treatments applied were identical to those used in the laboratory priming experiment. A total of 36 plastic pots with drainage holes were prepared. The soil, sourced from Kurdistan University’s research farm, was mixed in equal proportions (1:1:1) with sand and composted fertilizer. Before sowing, pots were saturated with water and allowed to drain to improve planting conditions. In each pot, 25 primed seeds were sown at a depth of 1 cm. The temperature inside the greenhouse was regulated by heating and cooling systems to maintain an optimal 25 °C throughout the growing season. Light and temperature conditions were closely monitored, and irrigation was carried out manually once a day. After 45 days, the seedlings were harvested. Shoots and roots from each treatment were collected separately, placed in paper bags, and dried at 70 °C for 72 h. The dry weights of the shoots and roots were then measured using a digital scale.

### Experiment 3 (field)

#### Farm Soil Characteristics

Prior to planting, random soil sampling was conducted from the experimental site. Subsequently, certain physical and chemical properties of the soil, such as soil texture, soil organic matter, soil nitrogen content, acidity (pH), and salinity (EC) were measured. Some physical and chemical properties of the soil at the experimental site are presented in the Table 1. The soil texture is clay loam.

**Table 1:** Soil Analysis Results of the Experimental Plot.

Soil texture	Clay (%)	Silt (%)	Sand (%)	Organic Matter (%)	pH	EC (dS.m^− 1^)	*N* (%)	K (mg.kg^− 1^)	*P* (mg.kg^− 1^)
Clay loam	29.30	40.23	30.47	1.21	7.65	0.82	0.10	450	16.20

#### Seed priming test in field condition

To validate the results obtained from the Petri dish and pot experiments, the selected priming treatments were evaluated under field conditions. The field experiment was carried out using a completely randomized block design with three replications at the research farm of Kurdistan University. This farm is situated in western Iran at approximately 35.18°N latitude, 46.35°E longitude, and an elevation of 1860 m above sea level. The region experiences a Mediterranean climate, characterized by cold, moist winters and dry, moderate summers.

To prepare the planting bed, soil preparation operations—including plowing, discing, and leveling—were carried out to improve soil structure. Six planting rows were established in the field, and seeds were sown at a rate designed to achieve a final plant density of 50 plants per square meter. The seeds were thoroughly covered with soil. The planting date was recorded as May 8, 2019. Irrigation was applied weekly using a sprinkler system. During the growing season, manual weeding was performed three times to ensure effective weed control. At the 5-leaf stage, thinning was conducted to reduce intraspecific competition and maintain the desired plant density of 50 plants per square meter. Following thinning, the field was uniformly fertilized with urea at a rate of 200 kg.ha^− 1^ and subsequently irrigated. To manage pests, particularly shoot borers, Imidacloprid pesticide was applied at the 4–5 leaf and tillering stages at a concentration of 15 cc per 20 L of water. Harvesting took place manually at physiological maturity on August 25, 2019, 109 days after planting. After harvesting, plants from each plot were dried under sunlight for ten days. At the end of the growing season, biological yield, grain yield, and harvest index were measured. The harvest index was calculated using the following formula: Harvest Index: After obtaining the biological yield and grain yield, the harvest index was obtained through Eq. 4.

(4) Harvest index= (economic yield)/(biological yield) × 100.

### Statistical methods

Data normality was assessed using Minitab software (Ver 16.2.0). Variance analysis was performed using SAS software (Ver9.1), the LSD test was used to compare the data means and graphs were generated using Microsoft Excel (Ver 2016).

## Results

### Experiment 1 (laboratory): seed priming test in Petri dishes

#### Germination Percentage

**Fig. 1:**
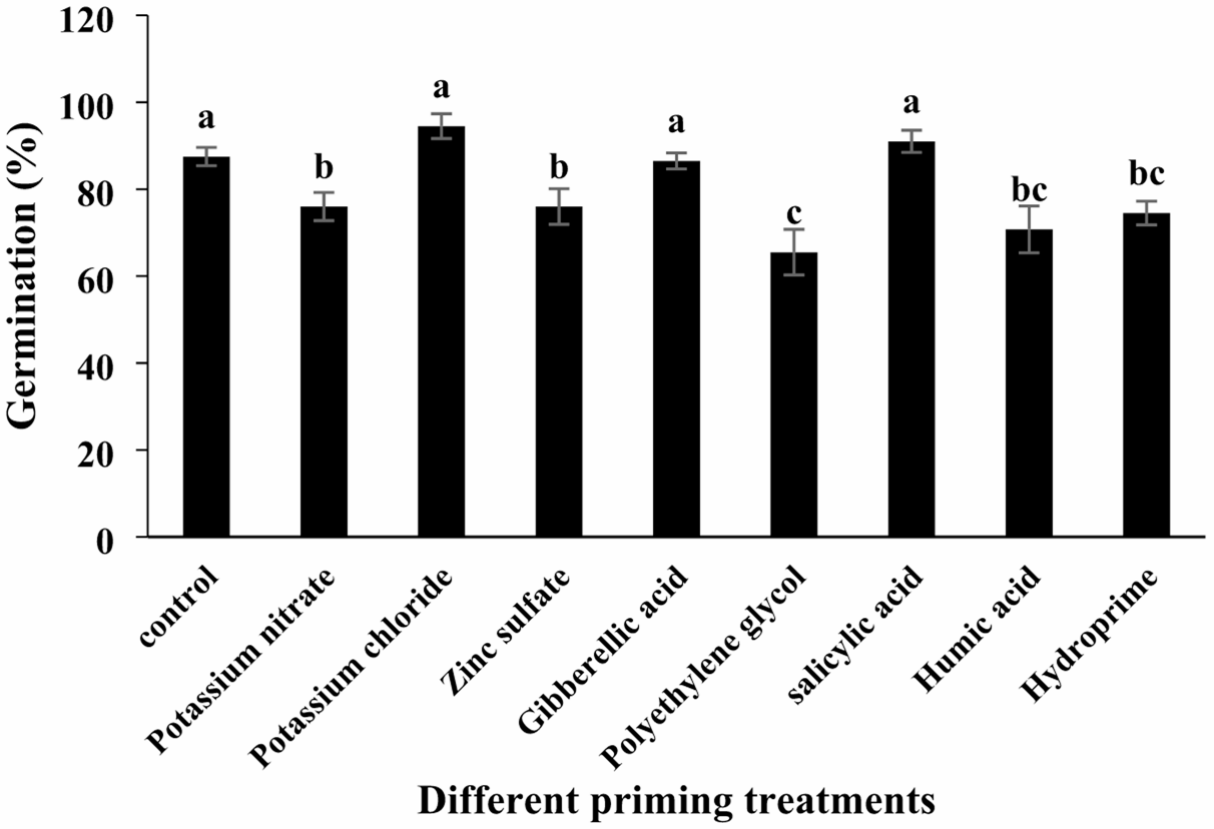
Effect of different seed priming treatments on quinoa germination percentage under laboratory conditions.

Analysis of variance revealed a significant effect of the different priming treatments on germination percentage (Table 2; *p* < 0.01). The highest germination percentage was observed in seeds primed with potassium chloride (94.5%), while the lowest was recorded for polyethylene glycol treatment (65.5%) (Table 3; Fig. 1). Potassium chloride priming increased germination by 8% compared to the control. In contrast, treatments with potassium nitrate, zinc sulfate, gibberellic acid, polyethylene glycol, salicylic acid, humic acid, and hydropriming resulted in germination percentage changes of -13%, -13%, + 1%, -25%, + 4%, -19%, and − 13%, respectively, relative to the control. Among these, only salicylic acid, gibberellic acid, and potassium chloride treatments showed increases in germination percentage compared to the control; however, these differences were not statistically significant.

**Table 2:** Analysis of variance for the effects of different seed priming treatments on germination characteristics of quinoa under Petri dish conditions.

Source of Variation	mean squares					
	df	Germination Percentage	Germination Rate	Mean Germination Time	Shoot Length	Root Length
Priming	8	393.62^**^	194.68^**^	0.094^*^	0.36^**^	0.47^**^
Error	27	50.50	61.66	0.020	0.069	0.052
Total	35	128.93	92.06	0.03	0.13	0.14
CV	-	8.85	11.87	9.44	4.51	5.72

ns, * and ** indicate non-significant, significant at the 5% level, and significant at the 1% level, respectively.

**Table 3:** Average comparison for the effects of different seed priming treatments on germination characteristics of quinoa under Petri dish conditions.

Treatment Priming	Germination (%)	Germination Rate (seeds per day)	Mean Germination Time (day)	Shoot Lenght (cm)	Root Lenght (cm)
Control (without priming)	87.50 a	69.24 abc	1.67 ab	5.50 d	3.70 ed
Potassium nitrate	76 b	63.10 dc	1.49 bcd	5.96 bc	4.63 a
Potassium chloride	94.50 a	77.84 a	1.53 bcd	6.35 a	4.15 bc
Zinc sulfate	76 b	63.35 bcd	1.43 cd	5.56 d	3.50 e
Gibberellic acid	86.50 a	74.71 ab	1.42 cd	5.70 cd	4.11 bc
Polyethylene glycol	65.50 c	55.88 d	1.41 cd	5.97 abc	3.90 cd
Salicylic acid	91 a	66.17 bcd	1.83 a	6.24 ab	4.24 b
Humic acid	70.75 bc	59.29 dc	1.54 ab	5.56 d	3.66 ed
Hydropriming	74.50 bc	65.71 bcd	1.33 d	5.85 cd	3.97 bcd

The values corresponding to the treatments examined in each column, which are marked with the same letters, do not have a statistically significant difference at the 5% probability level.

#### Germination Rate

**Fig. 2:**
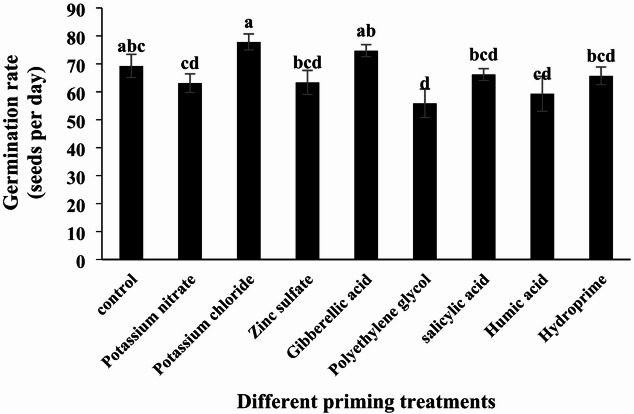
Effect of different seed priming treatments on the germination rate of quinoa under laboratory conditions.

Analysis of variance showed that different seed priming treatments had a significant effect on germination rate (Table 2; *p* < 0.01). The highest germination rate was observed with potassium chloride treatment (77.84 seeds per day), while the lowest was recorded for polyethylene glycol treatment (55.88 seeds per day) (Table 3; Fig. 2). The salicylic acid treatment resulted in an 8% decrease in germination rate compared to the control. Changes in germination rate relative to the control were as follows: potassium nitrate (-8.86%), potassium chloride (+ 12.42%), zinc sulfate (-8.50%), gibberellic acid (+ 7.90%), polyethylene glycol (-19.29%), humic acid (-4.37%), and hydropriming (-5.09%). While potassium chloride and gibberellic acid treatments enhanced the germination rate, these increases were not statistically significant. Among the treatments that reduced germination, only polyethylene glycol demonstrated a statistically significant decrease compared to the control.

#### Mean Germination Time (MGT)

**Fig. 3:**
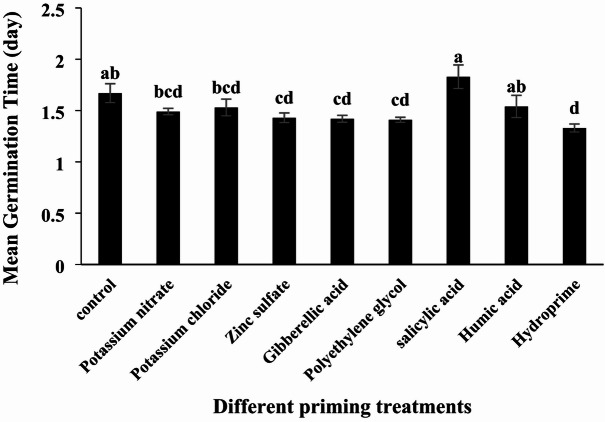
Effect of different seed priming treatments on the mean germination time of quinoa under laboratory conditions.

Analysis of variance indicated that the effect of different priming treatments on mean germination time (MGT) was significant (*p* < 0.05; Table 2). Potassium nitrate, humic acid, and potassium chloride treatments reduced MGT, although these reductions were not statistically significant compared to the control. Conversely, salicylic acid treatment was the only one to increase MGT, but this increase was also not statistically significant (Table 3; Fig. 3). Mean germination time is a key indicator of seed quality, representing the average time required for complete germination of a seed lot. A longer MGT implies delayed germination, reflecting lower seed vigor. Thus, MGT is inversely related to seed quality.

#### Shoot Length

**Fig. 4:**
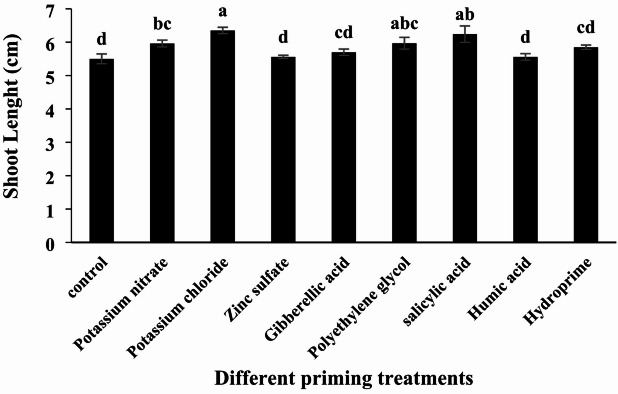
Effect of different seed priming treatments on quinoa shoot length under laboratory conditions.

According to the analysis of variance, different priming treatments had a significant effect on shoot length (Table 2, *p* < 0.05). The highest shoot length was observed in the potassium chloride treatment, measuring 6.35 cm, while the lowest was recorded in the untreated control at 5.50 cm. Potassium chloride treatment resulted in a 15% increase in shoot length compared to the control (Table 3; Fig. 4). Overall, potassium chloride, potassium nitrate, zinc sulfate, gibberellic acid, polyethylene glycol, salicylic acid, humic acid, and hydropriming treatments increased shoot length by 15%, 8%, 1%, 3%, 13%, 1%, and 6%, respectively, relative to the control. The potassium ions supplied during osmopriming play a crucial role in cellular potassium uptake and the activation of enzymes essential for germination and growth. Increasing shoot length in field conditions can enhance a plant’s competitiveness against weeds. Among the treatments, gibberellic acid and potassium chloride stand out as strong candidates, as they have positively influenced several parameters, including germination rate, mean germination time, and germination percentage (Figs. 1 and 2, and 3).

#### Root Length

**Fig. 5:**
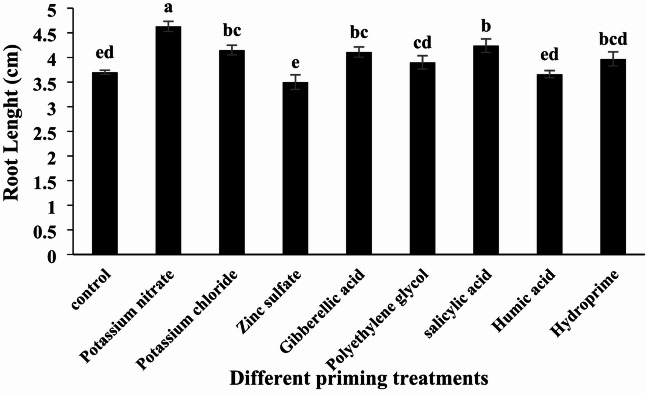
Effect of different seed priming treatments on quinoa root length under laboratory conditions.

According to the analysis of variance, different priming treatments had a significant effect on root length (Table 2, *p* < 0.05). The highest root length was observed in the potassium nitrate treatment, measuring 4.63 cm, while the lowest was recorded in the zinc sulfate treatment at 3.5 cm (Table 3; Fig. 5). Compared to the control, potassium nitrate, potassium chloride, zinc sulfate, gibberellic acid, salicylic acid, humic acid, and hydropriming treatments resulted in changes in root length of + 25%, + 12%, -5%, + 11%, + 5%, + 14%, and − 7%, respectively.

### Experiment 2 (greenhouse): seed priming test in pots

#### Shoot dry weight

**Fig. 6:**
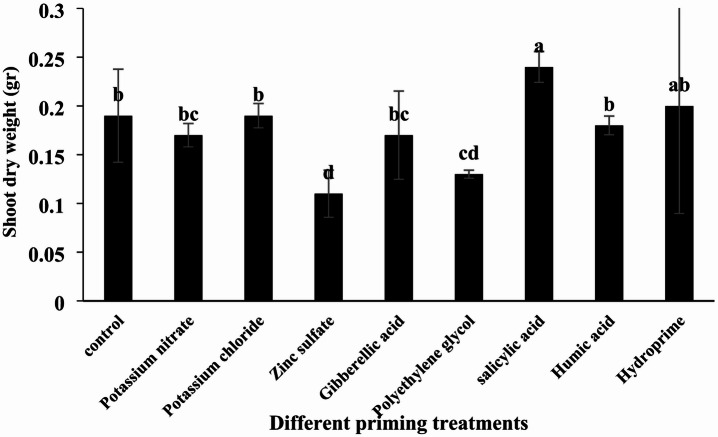
Comparison of the effects of different priming treatments on quinoa shoot dry weight under greenhouse conditions.

According to the analysis of variance, different priming treatments had a significant effect on shoot dry weight (Table 4, *p* < 0.05). The highest shoot dry weight was observed in the salicylic acid treatment (0.24 g per seedling), while the lowest was recorded in the zinc sulfate treatment (0.11 g per seedling). Although salicylic acid and hydropriming treatments increased shoot dry weight compared to the control, these differences were not statistically significant. Conversely, all other treatments resulted in a decrease in shoot dry weight relative to the control (Table 5; Fig. 6).

**Table 4:** Analysis of variance of the effects of different priming treatments on seedling characteristics of quinoa plants under greenhouse conditions.

Source of Variation	mean squares		
	df	Dry weight of shoot	Dry weight of root
Priming	8	0.0053^**^	0.00019^**^
Error	27	0.0012	0.000026
Total	35	0.0022	0.000062
CV	-	19.86	15.18

ns, * and ** indicate non-significant, significant at the 5% level, and significant at the 1% level, respectively.

**Table 5:** Comparing the average effects of different priming treatments on seedling characteristics under quinoa plants greenhouse conditions.

Treatment Priming	Shoot dry weight (gr)	Root dry weight (gr)
Control (without priming)	0.19 b	0.024 d
Potassium nitrate	0.17 bc	0.024 d
Potassium chloride	0.19 b	0.039 ab
Zinc sulfate	0.11 d	0.033 b
Gibberellic acid	0.17 bc	0.027 cd
Polyethylene glycol	0.13 cd	0.032 bc
Salicylic acid	0.24 a	0.045 a
Humic acid	0.18 b	0.037 ab
Hydropriming	0.20 ab	0.037 ab

The values ​​corresponding to the treatments examined in each column, which are marked with the same letters, do not have a statistically significant difference at the 5% probability level.

#### Root dry weight

**Fig. 7:**
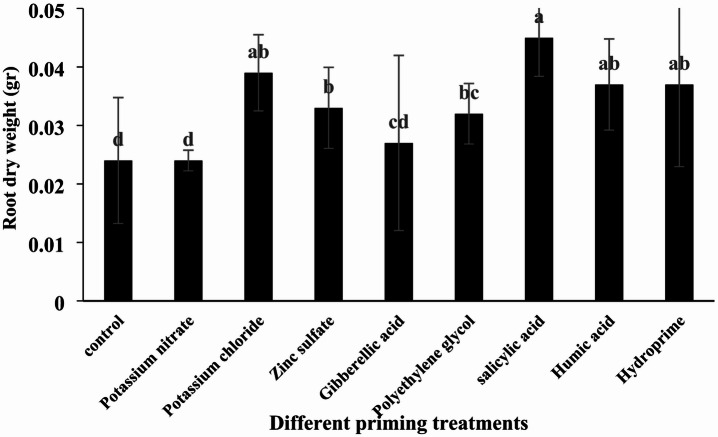
Comparison of the effects of different priming treatments on quinoa root dry weight under greenhouse conditions.

According to the original analysis of variance results, different priming treatments had a significant effect on root dry weight (Table 4, *p* < 0.05). The highest root dry weight was observed in the salicylic acid treatment (0.45 g per seedling), while the lowest values were recorded for potassium nitrate and control treatments (0.24 g per seedling). Treatments with potassium chloride, zinc sulfate, gibberellic acid, polyethylene glycol, hydropriming, and humic acid all increased root dry weight compared to the control (Table 5; Fig. 7).

### Experiment 3 (field): seed priming test in field condition

#### Biological yield

**Fig. 8:**
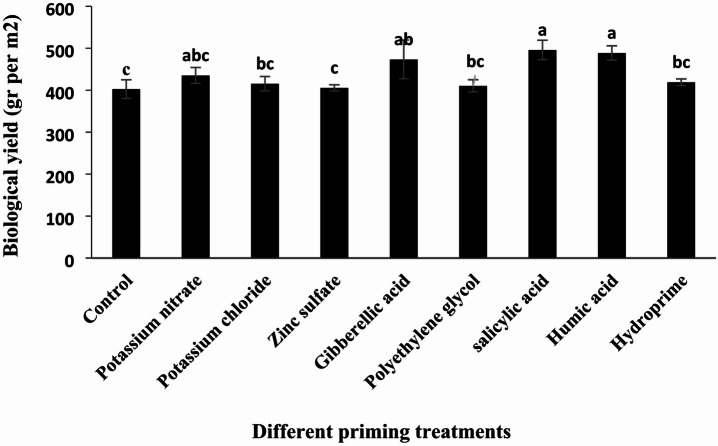
Comparison of the effects of different priming treatments on the biological yield of quinoa under field conditions.

According to the original analysis of variance, different priming treatments had a significant effect on biological yield (Table 6, *p* < 0.05). As shown in Table 7; Fig. 8, the highest biological yield was recorded in the salicylic acid treatment, with 496 g.m^−^², while the lowest yield was observed in the control treatment (without priming), at 402.5 g.m^−^². Compared to the control, the biological yield changes for potassium nitrate, potassium chloride, zinc sulfate, gibberellic acid, polyethylene glycol, salicylic acid, humic acid, and hydropriming treatments were + 8.11%, + 3.22%, + 0.78%, + 17.59%, + 1.98%, + 23.22%, + 21.42%, and + 4.08%, respectively.

**Table 6:** Analysis of variance of the effects of different priming treatments on yield traits of quinoa plants under field conditions.

Source of Variation	mean squares				
	df	Biological yield	Inflorescence yield	harvest index	Grain yield
Rep	2	2321.69 ns	1826.37 ns	229.96 **	4236.74 **
Priming	8	4171.73*	2018.19 *	47.36 **	942.89 *
Error	16	1376.51	696.02	7.64	318.06
Total	26	2309.28	1189.80	36.97	811.73
CV	-	8.75	8.39	12.09	8.39

ns, * and ** indicate non-significant, significant at the 5% level, and significant at the 1% level, respectively.

**Table 7:** Average comparison of the effects of different priming treatments on yield traits of quinoa plants under field conditions.

Treatment Priming	Biological yield (gr per m2)	Inflorescence yield (gr per m2)	Harvest Index (%)	Grain yield (gr per m2)
Control (without priming)	402.50 c	265.81c	47.45 cd	189.49 c
Potassium nitrate	435.17 abc	288.35c	46.38 d	203.04 bc
Potassium chloride	415.50 bc	302.30 abc	54.09 a	225.12 ab
Zinc sulfate	405.67 c	280.68 c	48.81 bcd	197.72 bc
Gibberellic acid	473.33 ab	300.88 bc	41.49 e	195.57 bc
Polyethylene glycol	410.50 bc	287.18 c	52.80 ab	215.35 abc
Salicylic acid	496 a	346.96 a	49.61 abc	245.45 a
Humic acid	488.75 a	336.53 ab	45.55 de	222.82 ab
Hydropriming	418.96 bc	303.23 abc	51.97 abc	218.05 abc

The values corresponding to the treatments examined in each column, which are marked with the same letters, do not have a statistically significant difference at the 5% probability level.

#### Inflorescence yield

**Fig. 9:**
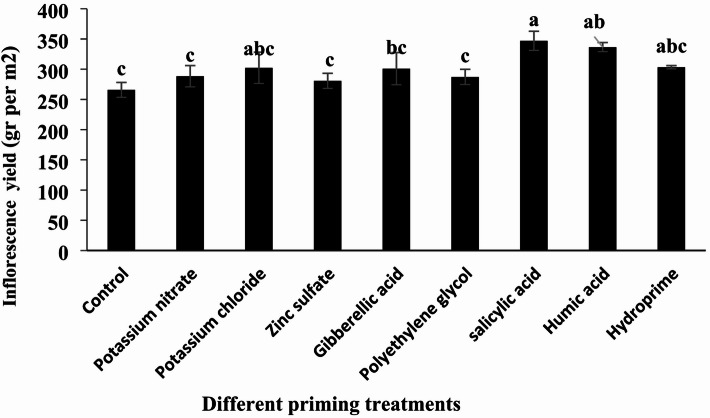
Comparison of the effects of different priming treatments on quinoa inflorescence yield under field conditions.

According to the analysis of variance, different priming treatments had a significant effect on inflorescence weight (Table 6, *p* < 0.05). The highest dry inflorescence weight was observed in the salicylic acid treatment, with 346.96 g.m^−^², while the lowest was recorded in the control treatment, at 265.81 g.m^−^². Compared to the control, the percentage changes in inflorescence weight for potassium nitrate, potassium chloride, zinc sulfate, gibberellic acid, polyethylene glycol, salicylic acid, humic acid, and hydropriming treatments were + 8.47%, + 13.72%, + 5.59%, + 13.19%, + 8.03%, + 30.52%, + 26.60%, and + 14.07%, respectively. All treatments had a positive effect on inflorescence weight, with salicylic acid and humic acid treatments showing the most pronounced impacts (Table 7; Fig. 9). However, the increases observed with zinc sulfate, potassium nitrate, and polyethylene glycol treatments were not statistically significant compared to the control.

#### Harvest index

**Fig. 10:**
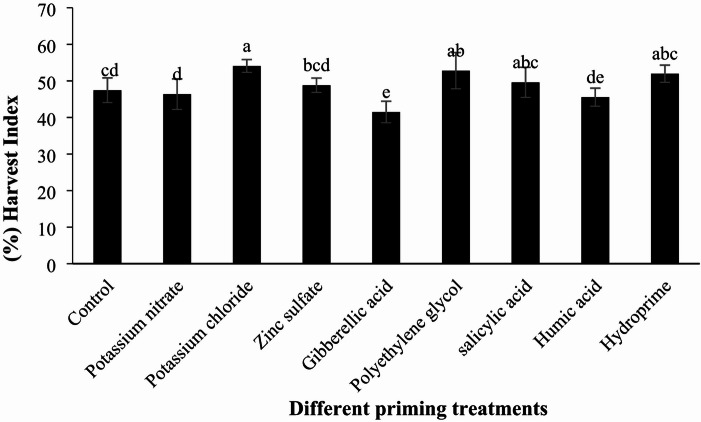
Comparison of the effects of different priming treatments on quinoa harvest index under field conditions.

According to the analysis of variance, the effect of different priming treatments on the harvest index was significant (Table 6, *p* < 0.01). The highest and lowest harvest indices were observed in the potassium chloride (109.54%) and gibberellic acid (49.41%) treatments, respectively. Compared to the control, the Harvest index changed by -2.25% (potassium nitrate), + 13.99% (potassium chloride), + 4.34% (zinc sulfate), -3.20% (gibberellic acid), + 11.27% (polyethylene glycol), + 29.53% (salicylic acid), + 17.58% (humic acid), and + 15.07% (hydropriming). Among these, potassium chloride, zinc sulfate, salicylic acid, polyethylene glycol, and hydropriming treatments improved the harvest index. However, only the differences between the potassium chloride and polyethylene glycol treatments and the control were statistically significant. In contrast, potassium nitrate, gibberellic acid, and humic acid treatments reduced the harvest index, with the reduction in the gibberellic acid treatment being significantly different from the control (Table 7; Fig. 10).

#### Grain yield

**Fig. 11:**
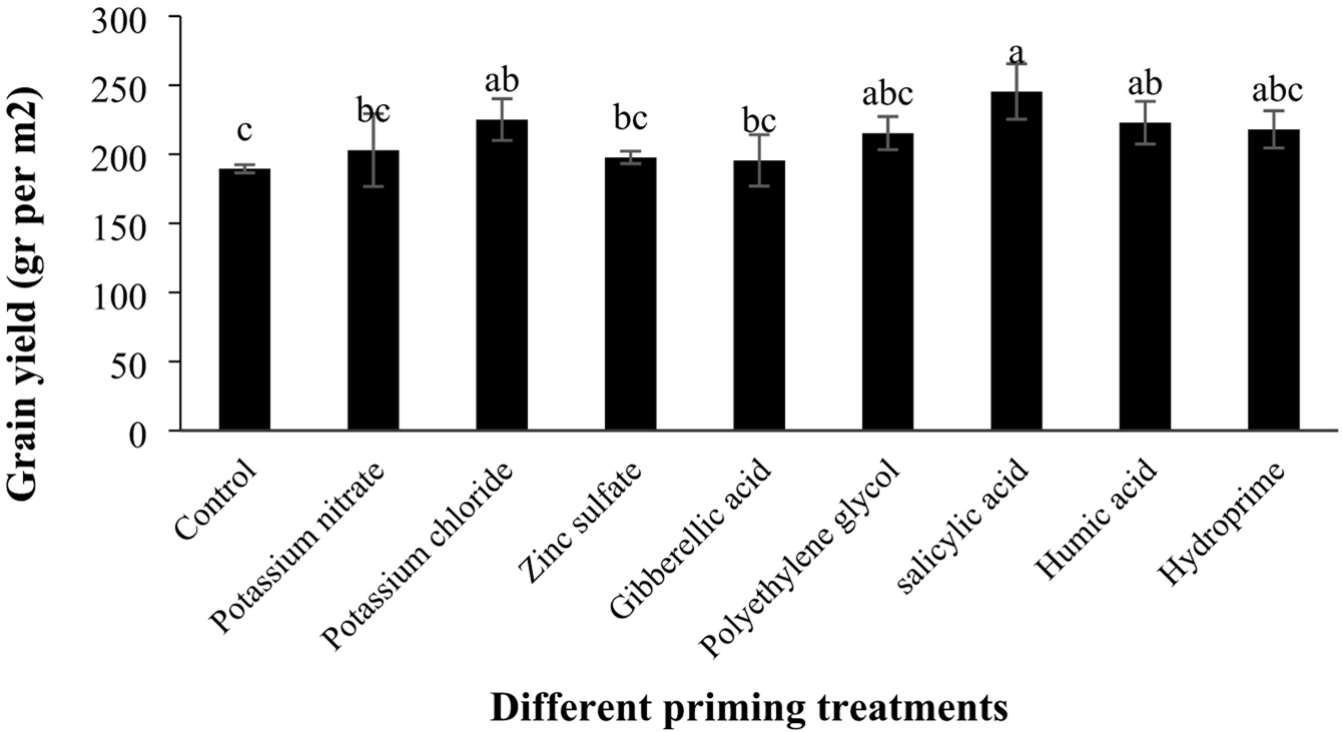
Comparison of the effects of different priming treatments on quinoa grain yield under field conditions.

According to the analysis of variance, the effect of different priming treatments on economic yield was significant (Table 6, *p* < 0.05). The highest economic yield was observed in the salicylic acid treatment (245.45 g.m^−^²), while the lowest was in the control treatment (189.49 g.m^−^²). Compared to the control, the changes in economic yield for potassium nitrate, potassium chloride, zinc sulfate, gibberellic acid, polyethylene glycol, salicylic acid, humic acid, and hydropriming treatments were + 7.15%, + 18.80%, + 4.34%, + 3.20%, + 13.64%, + 29.53%, + 17.58%, and + 15.07%, respectively (Table 7; Fig. 11).

### Correlation coefficients

The original correlation analysis results (Table 8) indicated several significant positive relationships among measured traits. Under laboratory conditions, seed germination percentage showed a significant positive correlation with germination rate (*p* < 0.01). Additionally, shoot length exhibited a significant positive correlation with root length (*p* < 0.05). Under greenhouse conditions, the dry weight of seedling shoots was significantly correlated with grain yield (*p* < 0.05). In field conditions, biological yield demonstrated a significant positive correlation with both grain yield and inflorescence yield (*p* < 0.01). Furthermore, grain yield was significantly and positively correlated with yield index and inflorescence yield (*p* < 0.01). These findings indicate that enhancements in early growth traits, such as shoot and root length or seedling dry weight, may lead to improved field performance and yield components.

**Table 8:** Correlation coefficients among measured traits of quinoa under laboratory, greenhouse, and field conditions in an experiment evaluating the effects of different priming treatments on germination characteristics, morphological traits, and yield.

x2	x1	x2	x3	x4	x5	x6	x7	x8	x9	x10
	0.875**	1								
x3	0.165^ns^	-0.314^ns^	1							
x4	0.236^ns^	0.220^ns^	-0.035^ns^	1						
x5	0.153^ns^	0.231^ns^	-0.151^ns^	0.544*	1					
x6	0.098^ns^	-0.010^ns^	0.300^ns^	0.123^ns^	0.237^ns^	1				
x7	0.262^ns^	0.221^ns^	0.049^ns^	0.356^ns^	-0.154^ns^	0.349^ns^	1			
x8	-0.090^ns^	-0.239^ns^	0.344^ns^	-0.085^ns^	0.132^ns^	0.189^ns^	-0.026^ns^	1		
x9	-0.171ns	-0.247^ns^	0.183^ns^	0.220^ns^	0.056^ns^	0.478*	0.326^ns^	0.468*	1	
x10	-0.094^ns^	-0.047^ns^	-0.095^ns^	0.317^ns^	-0.038^ns^	0.334^ns^	0.354^ns^	-0.343^ns^	0.663**	1
x11	-0.094^ns^	-0.186^ns^	0.226^ns^	0.123^ns^	0.110^ns^	0.339^ns^	0.238^ns^	0.820**	0.640**	0.001^ns^

**X1**,** X2**,** X3**,** X4**,** and X5** represent germination percentage, germination rate, average germination time, shoot length, and root length, respectively, under laboratory Petri dish conditions. **X6 and X7** denote shoot dry weight per seedling and root dry weight per seedling, respectively, under greenhouse pot cultivation. **X8**,** X9**,** X10**,** and X11** correspond to biological yield, grain yield, harvest index, and inflorescence yield, respectively, under field conditions. *ns*, *, and ** indicate non-significant, and significant at the 5% and 1% probability levels, respectively.

## Discussion

Seed priming has become an important technique for improving germination and growth traits in a variety of plant species, including quinoa. It has been reported^30^ that priming with potassium chloride enhances both physical and chemical activities within seeds, effectively reducing seed coat adhesion and facilitating root emergence without resistance. This observation aligns with the findings of previous research indicating that^31^ seed priming improves germination under saline stress by preventing cellular damage and inhibiting the absorption of harmful ions. In the context of quinoa seed priming, studies have found^17^ that gibberellic acid notably increased germination, while polyethylene glycol resulted in decreased germination rates, which is consistent with the results of the present study. However, a contrasting finding was noted regarding potassium nitrate treatment, which showed a decrease in germination percentage, differing from other work which documented^18^ an increase in germination percentage with potassium nitrate priming in quinoa seeds.

Furthermore it has been demonstrated^32^ that low concentrations of selenium priming enhanced quinoa seed germination. These discrepancies highlight that different quinoa genotypes may respond variably to priming treatments, emphasizing the need for genotype-specific approaches in priming protocols. The positive effects of seed priming on germination and growth characteristics of quinoa are evident, particularly when implemented and managed appropriately under field conditions, leading to improved crop performance. The favorable impact of potassium chloride on germination rate can be attributed to its osmotic benefits and its role as a cofactor in various enzyme activities, especially during radicle development and resource mobilization^33^. In contrast, treatment with polyethylene glycol (PEG) has been shown to decrease both the percentage and rate of germination. Previous studies suggest that excessive priming, even at optimal PEG concentrations, can disrupt α-amylase activity, leading to reduced germination rates and abnormal growth of shoots and roots. Additionally, the effectiveness of seed priming is affected by several factors, including the concentration of the priming solution, the duration of treatment, and the temperature^34,35^. Supporting these findings, research on barley has reported^36^ that priming with salicylic acid, gibberellic acid, and calcium chloride enhanced both germination percentage and rate. Similarly, evidence shows^37^ that saponin priming significantly improved quinoa seed germination rate. Overall, the results of this study suggest that treatments with gibberellic acid and potassium chloride effectively increase both germination percentage and rate, thereby identifying them as successful priming agents. Potassium enhances quinoa yield by improving seed germination, early seedling establishment, and tolerance to environmental stresses. This element ensures optimal plant density and robust early growth by increasing germination percentage, accelerating radicle and hypocotyl growth, and strengthening the antioxidant defense system, ultimately leading to enhanced grain yield and biomass. Studies indicate that optimal potassium application can significantly increase quinoa yield even under saline stress conditions. In addition to enhancing germination, previous studies have demonstrated that priming treatments reduce mean germination time (MGT) in quinoa seeds^37^.

This is corroborated by findings that^32^ low concentrations of selenium priming decreased MGT in quinoa. Uniform and rapid seedling emergence is crucial for achieving high-quality yields in field conditions. Priming, even if it does not significantly affect germination rates, can enhance plant growth by improving quality parameters like mean germination time (MGT). Recent studies have demonstrated that priming barley seeds with hydrogen peroxide, calcium chloride, salicylic acid, and succinic acid significantly increases both shoot and root length, particularly under low-temperature stress^38^. Similarly, studies on chickpea have reported^39^ that potassium chloride and hydrogen peroxide priming enhanced shoot and root length in chickpea plants under saline conditions. The increased root length observed in seeds primed with potassium nitrate may be attributed to faster germination, which promotes accelerated seedling growth and enhances both shoot and root development. Several studies have documented improvements in root and shoot length due to priming. For instance, reports indicate^40^ that seed priming with potassium nitrate increased shoot and root length in seedlings of various plant species under stress conditions. However, it is worth noting that other research has found^17^ that polyethylene glycol priming reduced root length in quinoa, which contradicts the findings of the present study and may be attributed to differences in experimental conditions or genotype responses.

Priming seeds under stress conditions has been shown to enhance the mobilization of seed reserves to the embryo, promoting better embryo growth and improving germination and related traits. This physiological process allows seedlings to utilize water and nutrients more efficiently, leading to greater growth compared to unprimed seeds. As a result, the dry weight of shoots and roots from primed seeds tends to increase^41^. It has been demonstrated^42^ that priming with humic acid, Pseudomonas, marlamin, and hydropriming led to increased shoot dry weight in barley under drought stress conditions. However, it is important to highlight that it has been reported^21^ a contrasting finding, indicating that hydropriming decreased shoot dry weight in quinoa under similar drought conditions. These varying outcomes indicate that although priming treatments typically boost seedling growth by enhancing the plant’s ability to absorb and utilize water and nutrients, the effectiveness of specific priming methods can vary based on the treatment type and plant species. This improved capacity for water and nutrient uptake through priming leads to greater dry matter accumulation in both shoot and root tissues. Supporting this notion, it has been reported^42^ that priming with humic acid, Pseudomonas, marlamin, and hydropriming significantly promoted root growth in barley subjected to drought stress. Similarly, it has been demonstrated^21^ that hydropriming increased root length in quinoa plants under drought stress, further indicating that priming can positively influence root development, which is critical for overall plant health and resilience.

Seed priming has been shown to significantly influence various aspects of plant growth and yield, contributing to enhanced biological yield and improved resource utilization. It has been attributed^43^ the increase in biological yield following priming to rapid plant establishment and more efficient resource utilization. This enhancement appears to be closely linked to improvements in seedling emergence percentage and rate. Similarly, it has been reported^44,45^ that the increase in biological yield due to priming can be associated with enhanced antioxidant activity, accelerated seedling growth, improved establishment, and more effective utilization of environmental factors such as light, soil moisture, and nutrients. Notably, it has been found^46^ that priming lentil seeds with zinc sulfate resulted in increases in grain and biological yields of up to 92%.

The impact of seed priming on chlorophyll activity and photosynthetic efficiency has also been documented. It has been indicated^47^ that seed priming influences chlorophyll activity, which subsequently enhances photosynthetic efficiency. This improvement is evident in the increased number of seeds per inflorescence after seed priming. This enhancement is due to improved photosynthetic activity resulting from earlier plant establishment and more efficient use of environmental resources^48^. Furthermore, the increased inflorescence weight resulting from seed priming is associated with improved germination and successful plant establishment. This enhanced early growth fosters vigorous vegetative development, which in turn contributes to better reproductive growth, including a higher inflorescence weight. Supporting this notion, it has been reported^49^ that priming with potassium chloride, calcium chloride, and potassium nitrate significantly increased inflorescence weight in rice plants, reinforcing the positive impact of seed priming on yield components. Despite the benefits associated with seed priming, the exact mechanisms behind the behavior of some priming treatments remain unclear. It has been reported^50^ an increase in the harvest index of chickpea seeds due to seed priming. Similarly, it has been found^51^ that priming with salicylic acid and zinc sulfate increased the maize harvest index by 24%.

It has been also reported^52^ improvements in the harvest index of wheat through priming with polyethylene glycol and hydropriming. Additionally, it has been observed^53^ an increase in the harvest index of quinoa plants following priming with potassium sulfate. Seed priming can also enhance plant growth rate, ultimately improving yield by shortening the greening period and reducing the base temperature required for germination^54^. It has been demonstrated^54^ that seed hydropriming can increase total chlorophyll content, including chlorophyll a and b, thereby enhancing photosynthetic activity and boosting organic matter production and nutrient availability. These physiological improvements contribute to increased crop performance. The overall enhancement in yield due to priming treatments can be attributed to faster and more uniform germination, rapid seedling growth, effective establishment, and better utilization of environmental and nutritional resources^55^. Further supporting this concept, it has been found^56^ that seed priming in spring canola improved both the quantity and quality of seed yield. Similarly, it has been reported^50^ that salicylic acid priming positively influenced chickpea yield. It has been demonstrated^57^ that priming mitigated the negative effects of salinity stress on wheat yield. Furthermore, it has been observed^27^ that priming—particularly hydropriming—increased sesame seed yield. It has been also reported^58^ a significant improvement in the economic yield of corn following seed priming with zinc sulfate.

Various seed priming treatments significantly influence the germination and growth parameters of quinoa, with potassium chloride and salicylic acid proving to be particularly effective. Priming with potassium chloride notably enhanced the germination percentage and rate, shoot length, and biological yield, likely due to its osmotic benefits and its role as a cofactor in enzymatic activities during early seedling development. Similarly, salicylic acid positively impacted several growth parameters, including the dry weight of shoots and roots, as well as economic yield, indicating its potential as a beneficial priming agent under suitable conditions. In contrast, treatments such as polyethylene glycol and potassium nitrate often led to reduced germination rates and growth, suggesting that priming protocols must be carefully tailored to optimize seed performance. The variability in treatment effectiveness observed in this and other studies highlights the necessity for genotype-specific priming strategies, as quinoa cultivars may respond differently based on their genetic makeup and environmental conditions. Overall, these findings suggest that seed priming is a promising, low-cost strategy to enhance the germination, growth, and yield performance of quinoa. This has practical implications for improving crop establishment and productivity, especially in suboptimal or stress-prone environments. Implementing effective priming techniques in agricultural practices could strengthen quinoa cultivation and contribute to food security and sustainable agricultural systems. Further research is recommended to refine priming protocols and elucidate their physiological and biochemical mechanisms, particularly under diverse environmental stresses.

## Conclusion

This study demonstrates that seed priming significantly enhances quinoa germination and yield. Potassium chloride (KCl) improved laboratory germination (94.5%) and shoot length (6.35 cm), while salicylic acid (SA) increased field biological yield (496 g.m^−^²) and grain yield (245.45 g.m^−^²). Conversely, polyethylene glycol (PEG) reduced germination (65.5%). Strong correlations between early growth traits and final yield suggest that optimized priming (e.g., KCl for germination, SA for yield) can boost quinoa productivity, particularly in stress-prone environments. Further research should refine protocols for diverse cultivars and conditions.

## Data Availability

The authors of this article are committed to ensuring that all data presented in this article was extracted based on sound scientific methods in the form of scientific experiments in an academic environment, and that all data is available for presentation upon request. The datasets generated and/or analyzed during this study are available from the corresponding author upon reasonable request.

## References

[1] Desa, U. *The World Population Prospects: 2015 Revision* (2015).

[2] Zulfiqar, F., Casadesús, A., Brockman, H. & Munné-Bosch, S. An overview of plant-based natural biostimulants for sustainable horticulture with a particular focus on moringa leaf extracts. *Plant Sci.* **295**, 110194 (2020). 32534612 10.1016/j.plantsci.2019.110194

[3] Ruiz, K. B. et al. Quinoa biodiversity and sustainability for food security under climate change. A review. *Agron. Sustain. Dev.* **34**, 349–359 (2014).

[4] Ruiz, K. et al. Quinoa–a model crop for understanding salt-tolerance mechanisms in halophytes. *Plant. Biosystems-An Int. J. Dealing all Aspects Plant. Biology*. **150**, 357–371 (2016).

[5] Bazile, D. et al. Worldwide Evaluations of Quinoa: Preliminary Results from Post International Year of Quinoa FAO Projects in Nine Countries. *Front. Plant. Sci.* **7**, 850 (2016). 27446101 10.3389/fpls.2016.00850 PMC4914551

[6] Asher, A., Galili, S., Whitney, T. & Rubinovich, L. The potential of quinoa (Chenopodium quinoa) cultivation in Israel as a dual-purpose crop for grain production and livestock feed. *Sci. Hort.* **272**, 109534 (2020).

[7] FAO. *Crops and livestock products*, (2023). https://www.fao.org/faostat/en/#data/QCL.

[8] Quinoa-Production-by-Country. World Population Review, (2026). Available online: https://worldpopulationreview.com/country-rankings/quinoa-production-by-country

[9] Bagheri, M. et al. Assessment of adaptability and seed yield stability of selected quinoa (Chenopodium quinoa Willd.) genotypes in spring cropping systems in cold and temperate regions of Iran. *Iran. Soc. Crops Plant. Breed. Sci.* **22**, 376–387 (2021).

[10] Salehi, M., Soltani, V. & Dehghani, F. Effect of sowing date on phenologic stages and yield of Quinoa (Chenopodium quinoa Willd.) under saline condition. *Environ. stresses crop Sci.* **12**, 923–932 (2019).

[11] Sepahvand, N. A. in *International Quinoa Conference 2016: Quinoa for Future Food and Nutrition Security in Marginal Environments Dubai, 6–8 December 2016.*

[12] Javed, S. A. et al. Interactive effect of different salinity sources and their formulations on plant growth, ionic homeostasis and seed quality of maize. *Chemosphere* **291**, 132678 (2022). 34710460 10.1016/j.chemosphere.2021.132678

[13] Marcos Filho, J. Seed vigor testing: an overview of the past, present and future perspective. *Scientia agricola*. **72**, 363–374 (2015).

[14] Al-Qabba, M. M. et al. Phenolic profile, antioxidant activity, and ameliorating efficacy of chenopodium quinoa sprouts against CCl4-induced oxidative stress in rats. *Nutrients* **12**, 2904 (2020). 32977429 10.3390/nu12102904 PMC7598205

[15] Nadali, F., Asghari, H., Dorostkar, V. & Bagheri, M. Physiological Responses of Quinoa Varieties (*Chenopodium quinoa* Willd) to Hydropriming and Drought Stress. *Isfahan Univ. Technology-Journal Crop Prod. Process.* **12**, 49–62 (2022).

[16] Nazih, A. et al. Effect of gibberellic acid and mechanical scarification on the germination and seedling stages of *Chenopodium quinoa* Willd. under salt stress. *Plants* **13**, 1330 (2024). 38794401 10.3390/plants13101330 PMC11125075

[17] Daur, I. Effects of hydro and hormonal priming on quinoa (Chenopodium quinoa willd.) seed germination under salt and drought stress. *Pak J. Bot.* **50**, 1669–1673 (2018).

[18] Mansouri, A. & Omidi, H. Effect of Chitosan Nano Particle and Potassium Nitrate on Germination and Some Morpho-physiological Characteristics of Seedlings of Quinoa (*Chenopodium quinoa*). *Iran. J. Seed Res.* **5**, 147–159 (2018).

[19] Gholami, S., Rostami, T., Ahmadi, K. & Bagheri, M. The effect of different concentrations of salicylic acid on germination characteristics of two genotypes of quinoa (Chenopodium quinoa willd.) under salinity stress. *Environ. Stresses Crop Sci.* **15**, 529–539 (2022).

[20] Mansouri, A. & Omidi, H. Effect of priming and seed age on germination, photosynthetic pigments, and biochemical content of Quinoa seedling. *Plant. Process. Function*. **11**, 243–260 (2022).

[21] Dashab, S. & Omidi, H. Effects of hydro-and bio-priming on some physiological and biochemical characteristics of quinoa (Chenopodium quinoa) seedlings under drought stress. *Iran. J. Plant. Physiol.* **11**, 3659–3682 (2021).

[22] Ali, L. G., Nulit, R., Ibrahim, M. H. & Yien, C. Y. S. Efficacy of KNO3, SiO2 and SA priming for improving emergence, seedling growth and antioxidant enzymes of rice (*Oryza sativa*), under drought. *Sci. Rep.* **11**, 3864 (2021). 33594103 10.1038/s41598-021-83434-3 PMC7887194

[23] Singh, V. P. et al. Role of salicylic acid-seed priming in the regulation of chromium (VI) and UV-B toxicity in maize seedlings. *Plant. Growth Regul.* **78**, 79–91 (2016).

[24] Eisvand, H., Alizadeh, M. & Fekri, A. How hormonal priming of aged and nonaged seeds of bromegrass affects seedling physiological characters. *J. New. Seeds*. **11**, 52–64 (2010).

[25] Khan, M. B., Gurchani, M. A., Hussain, M., Freed, S. & Mahmood, K. Wheat seed enhancement by vitamin and hormonal priming. *Pak. J. Bot.* **43**, 1495–1499 (2011).

[26] Shariatmadari, M. H., Parsa, M., Nezami, A. & Kafi, M. Effect of hydropriming on germination and growth indices in chickpea (Cicer arietinum L.) cultivars under drought stress in laboratory and glasshouse condition. *Iran. J. Seed Sci. Technol.* **7**, 243–256 (2018).

[27] Kazemi, K., Khajehosseini, M., Nezami, A. & Eskandari, H. The Effect of seed priming on germination, yield and the quality of sesame grains under deficit irrigation. *J. Crops Improv.* **18**, 373–388 (2016).

[28] Aveling, T. A. S. in *Global Perspectives on the Health of Seeds and Plant Propagation Material* (eds Maria Lodovica Gullino & Gary Munkvold) 17–28 (Springer Netherlands, 2014).

[29] Damalas, C. A., Koutroubas, S. D. & Fotiadis, S. Hydro-priming effects on seed germination and field performance of faba bean in spring sowing. *Agriculture* **9**, 201 (2019).

[30] Liu Jie, L. J., Liu GongShe, L. G., Qi DongMei, Q. D., Li FangFang, L. F. & Wang EnHua, W. E. Effect of PEG on germination and active oxygen metabolism in wildrye (*Leymus chinensis*) seeds. *Acta Prataculturae Sinica*. **11**, 59–64 (2002).

[31] Farhoudi, R., Sharifzadeh, F., Poustini, K. & Makkizadeh, M. Kochak Por, M. The effects of NaCl priming on salt tolerance in canola (Brassica napus) seedlings grown under saline conditions. *Seed Sci. Technol.* **35**, 754–759 (2007).

[32] Gholami, S. & Dehaghi, M. A. The effect of priming with different concentrations of selenium on germination indices of quinoa seeds and seedlings. *J. Crop Improvment*. **24**, 85–95 (2022).

[33] Farooq, M., Basra, S., Afzal, I. & Khaliq, A. Optimization of hydropriming techniques for rice seed invigoration. *Seed Sci. Technol.* **34**, 507–512 (2006).

[34] Savvides, A., Ali, S., Tester, M. & Fotopoulos, V. Chemical priming of plants against multiple abiotic stresses: mission possible? *Trends Plant Sci.* **21**, 329–340 (2016). 26704665 10.1016/j.tplants.2015.11.003

[35] Debbarma, A., Devi, J. & Barua, M. Seed priming durations and concentrations influence on germination and seedling growth of bitter gourd. *Vegetable Sci.* **45**, 137–139 (2018).

[36] Gins, E. Seed priming effects on seed quality and antioxidant system in the seedlings of *Amaranthus tricolor* L. *Quinoa Improv. Sustain. Prod.* **54**, 638–648 (2022).

[37] Moreno, C., Seal, C. & Papenbrock, J. Seed priming improves germination in saline conditions for *Chenopodium quinoa* and *Amaranthus caudatus*. *J. Agron. Crop. Sci.* **204**, 40–48 (2018).

[38] Feng, J., Gins, M. & Gins, V. Seed priming effects on morphological traits of *Amaranthus hypochondriacus* under optimal and low temperatures. *SABRAO J. Breed. Genet.* **54**, 649–658 (2022).

[39] Naz, F. et al. Effect of NaCl stress on Pisum sativum germination and seedling growth with the influence of seed priming with potassium (KCL and KOH). *American-Eurasian J. Agricultural Environ. Sci.* **14**, 1304–1311 (2014).

[40] Yagmur, M. & Kaydan, D. Alleviation of osmotic stress of water and salt in germination and seedling growth of triticale with seed priming treatments. *Afr. J. Biotechnol.* **7**, 2156–2162 (2008).

[41] Ansari, O., Azadi, M., Sharif-Zadeh, F. & Younesi, E. Effect of hormone priming on germination characteristics and enzyme activity of mountain rye (*Secale montanum*) seeds under drought stress conditions. *J. Stress Physiol. Biochem.* **9**, 61–71 (2013).

[42] Jalal, J., Razieh, K. & Edris, K. Improving of barley seedling growth by seed priming under water deficit stress. *J. Stress Physiol. Biochem.* **10**, 125–134 (2014).

[43] Aboutalebian, M. & rah chamandi ahmadvand, g. & jahedi, a. Effects of On-Farm Seed Priming and Sowing Date on Germination Properties and some Physiological Growth Indices of three Soybean Cultivars (*Glycine max* L.) in Hamedan. *Iran. J. Field Crop Sci.* **43**, 715–728 (2013).

[44] Afrouz, M. et al. Seed bio-priming with beneficial *Trichoderma harzianum* alleviates cold stress in maize. *PeerJ* **11**, e15644 (2023). 37645014 10.7717/peerj.15644 PMC10461543

[45] Hosseini-Moghaddam, M. et al. Seed coating with minerals and plant growth-promoting bacteria enhances drought tolerance in fennel (*Foeniculum vulgare* L). *Biocatal. Agric. Biotechnol.* **58**, 103202 (2024).

[46] Toklu, F. Effects of different priming treatments on seed germination properties, yield components and grain yield of lentil (*Lens culinaris* Medik). *Notulae Botanicae Horti Agrobotanici Cluj-Napoca*. **43**, 153–158 (2015).

[47] Abbas Dokht, H. & Arefbeyki, M. The effects of hydropriming, planting depth and nitrogen split application on grain yield and it’s components of 370 double cross hybrid corn in arid zone. *J. Plant. Prod. Res.* **22**, 149–172 (2015).

[48] Abbasdokht, H., Afshari, H., Owji, E. & Taheri, S. The effect of seed priming and different levels of nitrogen application on quantitative and qualitative yield of sunflower progress cultivar. *crop Physiol. J.* **8**, 105–120 (2016).

[49] Nazari, S., hossieni & Allahgholipour, M. The Effect of Seed Priming and Coating on Emergence Indices, Root Morphology and Phenological Stages of Two Rice Cultivars. *J. Crops Improv.* **25**, 1–16 (2023).

[50] Hassanzadeh Ghorttepe, A., Sharfi, S. & Abbasi Sadr, S. Effect of drought stress and seed priming on some vegetative and reproductive traits of castor bean (*Ricinus communis* l.) Var Esfahan. *Sci. Res. J. Ecophysiology Crop Plants (Agricultural Sciences)*. **12**, 75–88 (2018).

[51] Mahboob, W. et al. Seed priming improves the performance of late sown spring maize (Zea mays) through better crop stand and physiological attributes. *Int. J. Agric. Biology*. **17**, 491–498 (2015).

[52] Aboutalebian, M. A., Sharifzadeh, F., Jahansouz, M. R., Ahmadi, A. & Naghavi, M. R. The Effect of Seed Priming on Germination, Stand Establishment and Yield of Wheat (*Triticum aestivum* L.)Cultivars in Three Different Climates of Iran. *Iran. J. Field Crop Sci.* **39**, 145–154 (2008).

[53] Hassan, A., Hasnain, Z., Asadullah, M., Hussain, S. S. & Anees, M. A. Biological Response of Quinoa Plants to Various Nitrogen Levels and Priming Techniques. *Sarhad J. Agric.* **38**, 1510–1518 (2022).

[54] Roy, N. K. & Srlvastava, A. K. Adverse effect of salt-stress conditions on chlorophyll content in wheat (*Triticum aestivum*) leaves and its amelioration through pre-soaking treatments. *Indian J. Agricultural Sci.* **70**, 777–778 (2000).

[55] Subedi, K. & Ma, B. Seed priming does not improve corn yield in a humid temperate environment. *Agron. J.* **97**, 211–218 (2005).

[56] Khan, M. N. et al. Seed priming with gibberellic acid and melatonin in rapeseed: Consequences for improving yield and seed quality under drought and non-stress conditions. *Ind. Crops Prod.* **156**, 112850 (2020).

[57] Shaddad, M., El-Samad, A., Mostafa, D. & H. & Role of gibberellic acid (GA3) in improving salt stress tolerance of two wheat cultivars. *Int. J. Plant. Physiol. Biochem.* **5**, 50–57 (2013).

[58] Babaei, K., Tajbakhsh, M. & Siosemardeh, A. Effect of Priming and Sowing Date of Seed on Growth Indices of Plant and yield and Yield Components of seed of Maize Single Cross 260 (Fajr). *Plant. Prod. Technol.* **19**, 193–209 (2020).

